# Parent-directed intervention versus controls whilst their child waits for diagnostic assessment: a systematic review protocol

**DOI:** 10.1186/s13643-021-01615-7

**Published:** 2021-03-04

**Authors:** C. Bernie, M. Mitchell, K. Williams, T. May

**Affiliations:** 1 Department of Paediatrics, The University of Melbourne, Melbourne, Victoria Australia; 2 Department of Allied Health, The Royal Children’s Hospital, Melbourne, Victoria Australia; 3 The Murdoch Children’s Research Institute, Melbourne, Victoria Australia; 4 Department of Neurodevelopment and Disability, The Royal Children’s Hospital, Melbourne, Victoria Australia; 5 Department of Paediatrics, Monash University, Clayton, Victoria Australia; 6 Department of Developmental Paediatrics, Monash Children’s Hospital, Clayton, Australia

**Keywords:** Systematic review, Protocol, Waiting, Assessment, Diagnosis, Child, Parent, Caregiver, Intervention

## Abstract

**Background:**

Waiting lists are an ongoing issue for publicly funded community and hospital-based health services. Parents and caregivers are instrumental supports in the health and well-being of young and school-aged children, yet little is known about the way they can be supported during waiting periods. Given mounting evidence about the value of early intervention in physical and mental health literature, and waits for some public health services extending past 12 months, it is both timely and warranted to explore interim interventions that may be applied in this period.

**Methods:**

Intervention studies that have applied an educational programme, information, group-based support or individualised therapy to primary caregivers of children (heron referred to as parent-directed interventions), waiting for diagnostic assessment at any inpatient or outpatient health service and aged between 1 and 12 years of age, will be reviewed. These will include intervention studies of any type that have included more than 5 participants or participant groups and where a control or comparison group has been included. Abstract screening, full-text review, data extraction and risk of bias will be conducted by two reviewers. Relevant databases in health and education will be systematically searched using key words and Medical Subject Headings (MeSH) and grey literature will be explored. Databases will include PubMed, Ovid for MEDLINE and PsycINFO, EBSCO for the Cumulative Index of Nursing and Allied Health Literature (CINAHL), and the Education Resources Information Center (ERIC). Covidence© will be used to support abstract and full text screening, which will be completed by two main reviewers. Results will be tabulated, summarised and meta-analysed using a random-effects model, in any instance where concordant outcome measures have been applied. Results will be published and reported in line with PRISMA reporting guidelines.

**Discussion:**

Given little is known about effective support for families when children are awaiting diagnostic assessment for any medical, developmental or behavioural condition, the authors will synthesise existing evidence about parent-directed interventions in this period. It is hoped that by understanding the existing evidence interventions that are proven to be effective will be adopted and intervention innovation can occur.

**Systematic review registration:**

PROSPERO 2020 CRD42020159360

**Supplementary Information:**

The online version contains supplementary material available at 10.1186/s13643-021-01615-7.

## Background

For decades, family-centred care (FCC) is a philosophy that has gained momentum in paediatric healthcare and research [1, 2]. Bronfenbrenner’s ecological systems theory [3] and the World Health Organization’s International Classification of Functioning [4] have long described the link between family environment and an individual’s well-being. As an approach, however, FCC does not receive universal support [5, 6]. Despite observed evidence gaps, studies suggest factors such as parent-child attachment [7, 8], caregiver mental health [9, 10], resource access or socioeconomic status [11, 12], and health literacy or knowledge of caregivers [13, 14] can positively or negatively impact child health outcomes. Such findings continue to support the need for ongoing consideration of family-centred approaches when addressing child well-being in theory or practice.

One area of health-related disparity that continues to be explored is child and family access to healthcare services [15, 16]. As a construct, access to services has a number of dimensions including approachability, availability and affordability [16], where obstacles to access can include service cost, transportation limitations, and waiting times. Even in developed countries with well-resourced health care systems, waiting times for services remain a barrier to prompt receipt of care [17–20].

Researchers, clinicians and policy makers have sought to understand and address waiting list issues across a range of healthcare settings [21–24]. Issues, such as duration and variation of waiting times, and corresponding delays in service access, or service inequity, exist across diagnostic and intervention services in both public and private sectors, and typically occur when healthcare resources are allocated irrationally. For example being based on historical tertiary hospital allocations, rather than being based on statistical data justifying appropriate resource distribution [24, 25]. Across the National Health Service in the UK, one of the oldest public health system still in existence today, waiting lists have continued to impact on public perceptions and service quality since just after its inception [24, 25]. Referrers, service users, providers, policy makers and healthcare systems worldwide continue to be challenged by waiting lists despite innovation in approaches aimed at their reduction or eradication [25–27].

In paediatric healthcare, increased demand for assessment of neurological, cardiac, developmental and allergy-related conditions, at a secondary or tertiary service level, has been documented in recent years [28–31]. This has placed further pressure on paediatric public health services, who subsequently utilise waiting lists in the absence of, or in addition to, other strategies to manage demand. Waiting for a child’s assessment or access to intervention has been identified as a time of elevated parental stress by some researchers, and a period of “missed opportunities” for timely intervention by others. This period has subsequently been recognized as having the potential to negatively impact on the well-being of children and parents or caregivers of the child awaiting services [32–37].

To counteract the actual and possible negative effects of paediatric waiting periods, clinicians and researchers have begun to describe strategies to support parents, other caregivers and families in the interim [32, 38–40]. These strategies are directed to parents of children, rather than children themselves, in order to reduce parental stress, or increase parental knowledge and competence on the path to improving child and parent well-being. There remains no systematic review to the authors’ knowledge that has explored the efficacy of parent-directed interventions whilst their child waits for diagnostic or assessment-related service access. Preliminary searches suggest that there is paucity of studies in this area, hence the importance of exploring the true status of evidence relating to family support interventions during this stage of healthcare. Moreover, initial scoping of the literature has revealed significant variation in strategies that have been applied, ranging from information sheets to group-based intervention programmes [38–40]. Thus, the aims of this systematic review are:

1. To explore the nature of interventions implemented for or via parents or primary caregivers of children waiting for a diagnostic assessment
2. To evaluate the efficacy of parent-directed interventions compared with controls, for improving family health and service access outcomes, whilst their child waits for diagnostic assessment

## Methods

Methodology for this systematic review will be guided by the Preferred Reporting Items for Systematic Review and Meta-Analysis (PRISMA–P) [41] and the Cochrane Handbook for Systematic Reviews of Interventions Version 6.0 [42]. Abstract screening, full-text review and data extraction will be conducted by two independent reviewers. Disagreements that cannot be resolved at each of these stages will be referred to a third reviewer. All reviewing personnel have previous research and clinical expertise across different disciplines of paediatric healthcare. The Preferred Reporting Items for Systematic Review and Meta-Analysis Protocols (PRISMA-P) guidelines (Additional file 1) have supported the development of this protocol, which will further guide all actions taken by reviewers.

### Search strategy

Relevant databases for initial electronic searches were reviewed with library-based content experts and paediatric researchers with extensive systematic review publication experience. Moreover, the authors considered existing relevant literature and databases within which such studies were indexed. The following electronic databases were selected for comprehensive searching using subject or MeSH headings and keywords as relevant. Search terms and synonyms will be considered in relation to previously known papers of relevance and adapted to each database search as appropriate, within:

- PubMed
- MEDLINE (OvidSP)
- PsycINFO (OvidSP)
- CINAHL (EBSCO)
- ERIC (EBSCO)
- The Cochrane Central Register of Controlled Trials (CENTRAL, The Cochrane Library, latest issue)

Search terms will broadly address the concepts of referral or waiting, diagnosis or assessment, caregiving or parenting, childhood, and intervention or programme. We present the search strategy in Additional file 2. We will refine other search strategies according to other database key word trees and report them in the review.

To identify studies not otherwise indexed, we will search trial registries, repositories and reference lists of included articles, as well as grey literature that may be inclusive of brief reports, conference-related publications and masters and PhD theses/dissertations e.g. via ProQuest Dissertation & Theses (PQDT). We will contact experts in the field and authors of potentially relevant studies where required, which may lead to further study inclusions.

No publication year or language limits will be applied for this systematic review in the interests of comprehensiveness and broad applicability of results.

### Eligibility criteria and study selection

#### Design inclusion

Eligible study designs will include randomised controlled studies (RCTs) and non-randomised controlled studies, which have been peer-reviewed. The minimum requirement will be 5 or more participants, with a comparison group applied within the study. Such methods are selected to reduce risk of bias and ensure study conclusions/outcomes are considered in the context of usual or alternative paediatric care pathways. On the authors’ initial assessment, inclusion of RCTs only would be likely to result in a very low inclusion yield and would exclude studies using other controlled methodologies relevant to the objectives of the review. Where published papers include the same participants in their sample, the study with the largest sample size will be analysed. Table 1 presents inclusion and exclusion criteria for studies, with further descriptions detailed below.

**Table 1:** Inclusion and exclusion criteria

Concept	Inclusion criteria	Exclusion criteria
**Participant**		
Primary caregiver	The parent, primary caregiver or legal guardian of the child, including mother or father, foster parent, or other guardian, who provides primary and regular care to the child.	Sibling, grandparent or other relative who is not the primary care provider of the child.
Children aged 0–12	At least 75% of children in study below 13 years of age at the time of the diagnostic assessment or, where mean age of children is not included and median given, the median age below 12 years of age.	Children primarily above 12 years of age at the time of the diagnostic assessment. Study includes a 50–50 mix of children and adolescents Study does not involve children waiting for diagnostic assessment.
Waiting	Study period follows referral to, or registration with a health service, and is prior to diagnostic assessment.	Study period is primarily prior to referral or registration with health service. Study period follows diagnostic assessment.
Diagnostic assessment at a health service	Diagnostic assessment is any assessment where an aspect of a child’s mental, physical, behavioural or developmental status or well-being is being assessed for the purpose of a diagnosis. Health service is any inpatient our outpatient health service, hospital or community-based, and may include mental health services, general practitioner services, secondary or tertiary team or individual assessments.	An assessment which is primarily for the purpose of therapy provisions only. An assessment which follows a diagnosis.
**Intervention**		
Parent-directed	An intervention directed to a parent or primary caregiver as defined above.	Intervention is not directed to the parent or primary caregiver, e.g. child-directed interventions or sibling group programmes, or parent training so they can deliver an intervention to their child.
Type	Intervention that targets knowledge, behaviour or actions and may include information provision, individualised or group-based education programmes, online programmes, face-to-face therapies, telephone or teleconference-delivered interventions.	Medical device or pharmaceutical-related intervention
**Comparator**		
Usual care	Any defined usual care provision, including waiting on waiting list, or measured lag between referral and initial diagnostic assessment	Usual care that does not occur in conjunction with a waiting period prior to a diagnostic assessment, as defined above, including care that occurs prior to the referral, or after the diagnostic assessment.
Other intervention	Any child- or parent-directed intervention occurring whilst waiting for diagnostic assessment, as listed above.	Any intervention that does not occur in conjunction with a waiting period prior to a diagnostic assessment, as defined above, including interventions that occur prior to the referral, or after the diagnostic assessment.
**Outcome**		
Primary outcome	Any	None
Secondary outcome	Any	None

Details of inclusions and exclusions are provided in Table 1. In brief, we will include all studies that evaluate interventions directed at parents or primary caregivers of children below 13 years of age. Strategies that are part of the intervention could address goals relating to the parent or primary caregiver, the referred child, siblings or the broader family as a unit. Children must be referred to or currently be waiting for a health-related diagnostic service. The assessment for diagnosis may be related to any medical, developmental or psychiatric condition. Subgroup data will be sought directly from authors where divisions are unclear and where appropriate for analysis. If no response is received, the paper will be excluded from further analysis.

#### Study selection process

The first author will search all databases listed above. Retrieved articles will be stored in Endnote©. Duplicates will be removed via manual review and automatic functions contained within Endnote, and these numbers will be recorded. Remaining articles will then be uploaded into Covidence© with any additional duplicates removed as identified by this software. These yield numbers will be recorded and formulate the beginning of the PRISMA flow chart for later publication with the main review manuscript.

The first and second authors will independently screen all titles and abstracts identified from searches in Covidence©, filtering out those that do not meet the inclusion criteria. We will retrieve the full text of any papers identified as potentially meeting the criteria by at least one author. Two review authors will then independently screen full-text articles for inclusion or exclusion, with discrepancies resolved by discussion. When consensus cannot be reached, a third author will be consulted to reach a decision.

Full-text papers excluded from the review will be listed as excluded studies with reasons provided in a ‘Characteristics of excluded studies’ table. We will also provide citation details and any available information about ongoing studies and consider details of duplicate publications, so that each investigation (rather than each report) is the unit of interest in the review. We will report the screening and selection process in an adapted PRISMA flow chart [41].

### Data extraction

Two review authors will extract data independently from included studies, using a custom designed electronic data extraction form in Microsoft Excel©. Any discrepancies in relation to data extraction will be resolved by discussion until consensus is reached or through consultation with a third reviewer if necessary. We will develop and pilot a data extraction form using the Cochrane guidance. This will be trialled on 3 papers, with relevant adjustments made, prior to its application in the extraction of data from all other papers.

Data to be extracted using categories detailed in Table 2.

**Table 2:** Data extraction categories

Study details	Participant characteristics	Methods	Intervention	Outcome examples (further detail provided in Table 3)
Authors, journal, year of publication, year recruitment began, country, study aims and objectives	Child gender, child age, child school participation status, primary caregiver type, gender, age, education level, other demographics relevant to study and review.	Design, allocation, sampling, blinding, data collection time points, loss to follow up, recruitment and retention rates, comparison/ control group	Setting: location, environment, technology used if relevant Description: intervention type, duration, frequency of intervention contacts.	Primary outcomes: Primary caregiver and/or family health outcomes, such as parenting stress or family quality of life Secondary outcomes: Child-related outcomes, such as child adaptive behaviour, quality of life and service-related outcomes, such as adherence to first appointment. These outcomes are of potential interest to researchers, policy-makers and clinicians

In the case of missing data, the contact authors for the study will be contacted via email for further information.

All extracted data will be entered into the extraction form in Microsoft Excel by the first author and will be checked for accuracy against the data extraction sheets of a second reviewer working independently. Conflicts and inconsistencies will be resolved jointly or with support of a third reviewer where consensus is unable to be reached. Possible outcomes of interest to the authors are detailed in Table 3.

**Table 3:** Primary and secondary outcomes of interest

Outcome category	Constructs	Measures and time-points
**Primary outcomes**		
Parent and family related outcomes	Parenting stress	Parent-reported questionnaire e.g. the Parenting Stress Index (normative standard score) A continuous variable, measured pre and post intervention/waiting period or post assessment appointment.
	Family quality of life	Parent Questionnaire e.g. The Family Quality of Life Scale (normative score) A continuous variable, measured pre and post intervention/waiting period or post assessment appointment.
**Secondary outcomes**		
Child-related outcomes	Child adaptive behaviour/global functional skills	Parent-reported questionnaire e.g. the Vinelands Adaptive Behaviour Scale (normative standard score) A continuous variable, measured pre- and post intervention/waiting period or post assessment appointment.
Service-related outcomes	Time to assessment	Time in days, weeks or months A continuous variable measured post assessment appointment.
	Adherence or attendance	Attendance or adherence to recommendations achieved or not achieved. A dichotomous variable measured post assessment appointment.

### Adverse events

Serious adverse events are not expected in studies of interest to this systematic review. An increase in parenting stress or decrease in family quality of life following an intervention will be considered an adverse event for the purposes of analysis and discussion.

### Data synthesis

We will decide whether to meta-analyse data based on whether the interventions in the included trials are similar enough in terms of participants, settings, intervention, comparison and outcome measures to ensure meaningful conclusions from a statistically pooled result. Where possible, we will standardise the data and generate pooled estimates, using the latest available version of Stata. For dichotomous data, we will use Stata to calculate odds ratios using inverse variance (IV) to produce a pooled estimate and explore random effects, with a 95% confidence interval. For continuous data, we will also use IV to explore the standardised mean difference, where pre- and post means and standard deviations are provided, again with a 95% confidence interval. We will visualise data for each outcome using forest plots and calculate heterogeneity. Forest plots will be created in Stata, with odds ratios or standard mean differences, weights and 95% confidence intervals. Using both visual inspection and the chi^2^ test for heterogeneity, further decisions will be made regarding meta-analyses. The *I*^2^ statistic will be used to quantify heterogeneity, with values of 50% or more representing levels that may preclude further meta-analysis. This will be considered within the context of the *p* value of the chi^2^ test, as well as the size and direction of the effects. If heterogeneity is high, we will conduct sensitivity analyses for methodological differences to explore the cause. We will present information about the impact of sensitivity analyses on heterogeneity and make a decision about presenting subgroup forest plots and conducting subgroup or overall meta-analyses on an individual outcome basis. We will conduct sensitivity analyses to assess heterogeneity and effect size comparing non-randomised and randomised studies and for randomised studies only. We will present randomised and non-randomised trials as subgroups in the same forest plot and only provide an overall effect size if heterogeneity is similar with and without non-randomised studies.

If we are unable to pool the data statistically using meta-analysis, we will provide clear reasons for this decision and will conduct a narrative synthesis of results. We will present the major outcomes and results, organised by intervention categories according to the major types and/or aims of the identified interventions. Depending on the assembled research, we may also explore the possibility of organising the data by the service category that the child is waiting for. Within the data categories, we will explore the main comparisons of the review:

- Intervention versus a control group or usual care.
- One form of intervention versus another.

Where studies compare more than one intervention, we will compare each separately to no intervention/control and with one another.

We will consider the following factors for subgroup analysis should this be possible:

1. Participant characteristics: Gender of participants, group vs individual intervention focus, age of child, type of service not yet provided.
2. Intervention: Study methodology used, type of intervention.
3. Primary outcome type (see Table 2).

Where qualitative data is presented, key themes will be collated in this review.

### Data quality and risk of bias

We will assess and report on the methodological risk of bias of included studies in accordance with the Cochrane Handbook [42], which includes recommendations around the explicit reporting of the following individual elements: random sequence generation, allocation sequence concealment, blinding (participants, personnel), blinding (outcome assessment), completeness of outcome data and selective outcome reporting. We will consider blinding separately for different outcomes where appropriate (for example, blinding may have the potential to differently affect subjective versus objective outcome measures). For non-randomised studies, we will also consider assignment of patients to treatment groups, the timing of intervention versus control groups, comparable baseline characteristics and loss to follow-up. We will judge each item as being at high, low or unclear risk of bias, and provide a quote from the study report and a justification for our judgement for each item in the risk of bias table.

In all cases, two authors will independently assess the risk of bias of included studies, with any disagreements resolved by discussion or referred to a third review in instances where consensus is unable to be reached. We will contact study authors for additional information about the included studies or for clarification of the study methods as required. We will incorporate the results of the risk of bias assessment into the review through standard tables, and systematic narrative description and commentary about each of the elements, leading to an overall assessment the risk of bias of each included study. As randomised and non-randomised studies will be included, it is likely that high levels of bias will be encountered and evaluated accordingly.

We will assess reporting bias qualitatively based on the characteristics of the included studies (e.g. if only small studies that indicate positive findings are identified for inclusion and if information that we obtain from contacting experts and authors or studies suggests that there are relevant unpublished studies).

If we identify sufficient studies for inclusion in the review, we will construct a funnel plot to investigate small study effects, which may indicate the presence of publication bias. We will formally test for funnel plot asymmetry, bearing in mind that there may be several reasons for funnel plot asymmetry when interpreting the results. We will conduct sensitivity analyses based on study quality.

### Confidence in evidence and representation

We will prepare a 'Summary of findings' table to present the results of meta-analysis and/or narrative synthesis for the major comparisons of the review, for each of the primary outcomes listed in Table 2. We will provide a source and rationale for each assumed risk cited in the table(s) and will use the Grading of Recommendations Assessment, Development and Evaluation (GRADE) criteria to rate the evidence based on the methods described in chapter 11 of the Cochrane Handbook [42]. If meta-analysis is not possible, we will present results in a narrative ‘Summary of findings’ table format.

## Discussion

Despite initiatives to reduce waiting times across particular programmes, extended waiting periods that can last months or years continue to plague some public health assessment services. Perils relating to waiting lists and health service access issues continue to be reported as an area of concern in paediatric research. This systematic review aims to evaluate applied interventions that are directed at parents of children who are awaiting diagnostic assessments, when compared with controls.

The strengths of this review include the novel nature of the topic, its use of relevant systematic review guidelines such as PRISMA to support a standardised approach to the review’s methods, well-defined inclusion and exclusion criteria, and its breadth of inclusion relating to health services and settings where parent-directed interventions for children waiting for diagnostic assessment may occur. There will be possible challenges and potential limitations for the evidence synthesis, given variable interventions and outcomes that may be yielded in the search. Despite these challenges, this review aims to inform authors, and the wider community about the reported value of interventions currently provided to parents of children awaiting diagnostic assessment. The outcomes of this review will detail the range of interventions that are effective for improving family health and service-related outcomes whilst families wait for diagnostic assessment services for their child. This information will be useful for clinicians, service providers and policy makers, so that there may be consideration of implementation of effective waitlist interventions in existing services. It will also highlight any research gaps in this area, and authors will propose opportunities for innovation and future directions based on the evidence synthesised.

## Supplementary Information

**Additional file 1:.** Prisma P Checklist.

**Additional file 2:.** Search Strategy.

## Data Availability

Not applicable

## Abbreviations

CENTRAL: Cochrane Central Register of Controlled Trials
CINAHL: Cumulative Index of Nursing and Allied Health Literature
EBSCO: Elton B. Stephens Co
ERIC: Education Resources Information Center
GRADE: Grading of Recommendations Assessment, Development and Evaluation
MEDLINE: A bibliographic database of life sciences and biomedical information
MeSH: Medical Subject Headings
OvidSP: Research platform
PQDT: ProQuest Dissertation & Theses
PRISMA: Preferred Reporting Items for Systematic Reviews and Meta-Analyses
PRISMA-P: Preferred Reporting Items for Systematic Reviews and Meta-Analyses–Protocol extension
PROSPERO: International prospective register of systematic reviews
PsycINFO: Abstracting and indexing database covering behavioural sciences and mental health
PubMed: Biomedical and life sciences journal literature
RCT: Randomised control trial
