# Supplementary material for: Parent-directed intervention versus controls whilst their child waits for diagnostic assessment: a systematic review protocol
Source: Syst Rev. 2021 Mar 4;10:67. doi: 10.1186/s13643-021-01615-7 (PMC7931343; doi:10.1186/s13643-021-01615-7)
Supplement: Supplementary file 2 — Additional file 2:. Search Strategy. [file 13643_2021_1615_MOESM2_ESM.docx]

| Population |  | Intervention | Outcome/  Comparators | Limits |
| --- | --- | --- | --- | --- |
| Age Group | Diagnoses | Intervention/Issues | Outcome | Limits |
| Primary Caregivers of children 0-12 | Awaiting an assessment for the purpose of diagnosis | Therapy programme or intervention (group or individual)    Information programme (face to face, telephone, video, handout, other) | To ensure comprehensiveness, search will not be limited by outcome or comparators, though screening will ensure a comparison group is identified in the abstract/study. | Nil |

APPENDIX B

“Primary caregiver-directed interventions versus controls whilst their child waits for diagnostic assessment: a systematic review protocol”

GENERAL AND EXAMPLE SEARCH STRATEGY

Systematic Review – General Search Strategy

Example Search Strategy (Ebsco – CINAHL)

| 1 | MH "Parents" OR MH “Caregiver” OR “carer*” OR “parent*” |
| --- | --- |
| 2 | MH "Fathers" OR MH "Mothers" OR MH "Family" |
| 3 | MH "Child" OR "child*" |
| 4 | MH "Waiting Lists" OR "wait*" |
| 5 | MH "Referral and Consultation" OR “refer*” |
| 6 | MH "Health Services Administration" OR MH "Management" |
| 7 | MH "Patient Education" OR MH "Health Education" OR MH "Parenting Education" |
| 8 | MH "Early Childhood Intervention" OR MH "Early Intervention" "OR "intervention*" OR “therap* OR "program*” OR |
| 9 | MH "Intervention Trials" OR MH "Clinical Trials” |
| 10 | (1 OR 2) AND 3 AND (4 OR 5 OR 6) AND (7 OR 8 OR 9) |
